# Editorial: Paradigm Changes are Required in HIV Vaccine Research

**DOI:** 10.3389/fimmu.2015.00326

**Published:** 2015-06-23

**Authors:** Marc H. V. Van Regenmortel

**Affiliations:** ^1^CNRS, UMR 7242 Biotechnologie et Signalisation Cellulaire, Institut de Recherche de l’Ecole de Biotechnologie de Strasbourg (IREBS), Ecole Supérieure de Biotechnologie Strasbourg (ESBS), University of Strasbourg, Illkirch, France

**Keywords:** antibody affinity maturation, paradigms in vaccine research, rational HIV-1 vaccine design, reverse vaccinology, vaccine immunogenicity

In his influential book “The Structure of Scientific Revolutions” published in 1962, Thomas Kuhn argued that researchers in every field of scientific enquiry are always guided by theoretical assumptions, presuppositions, and hypotheses that constitute the prevailing scientific paradigm under which they operate at any given time (1). When they operate within a shared paradigm, a scientific community working in a particular field will be committed to using certain strategies and experimental approaches that are accepted as being essential for trying to solve the problem at hand.

In many cases, the implicit hypotheses and presuppositions that give rise to a particular paradigm are not clearly stated and investigators may therefore sometimes not even be aware of them. One consequence of this is that when they obtain results that are not compatible with the theory and hypotheses underlying the paradigm, they may fail to appreciate that their guiding paradigm has in fact been refuted and should therefore be abandoned or revised. On the other hand, according to Kuhn, scientists actually do not abandon their hypotheses and paradigms as soon as contradictory data are obtained, since their main goal is not to try to confirm the validity of the underlying assumptions of their paradigms. Scientific communities tend to remain committed to their shared theoretical beliefs even when they obtain anomalous results that are not consistent with the paradigm, and they will invent new *ad hoc* hypotheses in an attempt to resolve apparent contradictions between theory and experimental observations (2). Unfortunately, when misleading paradigms are not discarded, this may encourage scientists to pursue unfruitful lines of investigations that could impede scientific progress.

In the field of HIV vaccine research, there is in fact evidence that in recent years a number of paradigms based on invalid assumptions had such a detrimental effect (3). This drawback can only be avoided if scientists keep in mind that if the paradigms they have adopted are based on erroneous assumptions, this could lead them to select inappropriate research strategies that are unlikely to succeed. Questioning the validity of paradigms is thus an important safeguard since it may reveal which invalid assumptions have led investigators astray in the past. Only when past mistakes are acknowledged can novel paradigms be introduced, which better fit our improved knowledge of HIV-1 immune responses, and are therefore more likely to help future vaccine development (4, 5).

The following unwarranted assumptions underlying some popular paradigms in HIV vaccine research may have contributed to our inability during the past 25 years to develop an effective HIV-1 vaccine.

## Assumption No. 1: Vaccine Immunogenicity can be Predicted from Viral Antigenicity

Most fragments of a viral antigen are immunogenic and are able to induce antibodies that will react with the fragment. However, this type of immunogenicity is irrelevant for vaccination since these antibodies rarely recognize the cognate, intact antigen, and even more rarely neutralize the infectivity of the viral pathogen that harbors the antigen (6). A confusion between biological immunogenicity (the ability to induce antibodies in a host) and chemical antigenicity (the capacity of an antigen to bind antibodies) lies at the heart of the reverse vaccinology paradigm that has been pursued vigorously for more than 10 years. According to this paradigm, HIV-1 epitopes identified by X-ray crystallographic analysis of complexes of HIV Env bound to affinity-matured neutralizing monoclonal antibodies (nMabs) were expected to be also effective vaccine immunogens able to induce a protective immune response. However, an epitope that binds to a nMab will not necessarily be able to induce the same type of neutralizing antibody in an immunized host. As discussed at length elsewhere (7), investigators who claim they are designing a vaccine immunogen are only improving the binding reactivity (i.e., the antigenicity) of a single epitope–paratope pair and are not actually designing a vaccine immunogen able to generate protective antibodies. Immunogenicity does depend on numerous factors that exist only in the context of the host immune system, and these are independent of the binding properties of the viral antigen used for immunization (8).

## Assumption No. 2: There is a Primary and Intrinsic Epitope Specific for Each B Cell Receptor and its Corresponding Antibody

In reality, there is no single intrinsic epitope for any antibody molecule but only a diverse group of potential ligands able to bind to it with various degrees of fit (3). Vaccinologists have been slow to accept that antibodies are not monospecific for a single epitope and that the degeneracy of the immune system always makes antibodies polyspecific for numerous related or unrelated epitopes present in different antigens (9–11). Once it is accepted that the epitope identified by X-ray crystallography of an HIV-1 Env-nMab complex is only one of the many epitopes that could be accommodated by that antibody, there is no justification for assuming that this particular HIV-1 epitope must correspond to the immunogen that elicited the nMab and should therefore be considered as a possible candidate vaccine.

## Assumption No. 3: HIV-1 Epitopes Recognized by Mature nMabs Isolated from HIV-1 Infected Individuals after a Lengthy Process of Antibody Affinity Maturation will be able to Induce a Protective Immune Response in Naive Individuals

It has been established that the initial HIV-1 immunogen that triggers the affinity maturation process leading to mature neutralizing antibodies usually recognizes a BCR germline version that differs considerably from the BCRs corresponding to mature antibodies (12). The very extensive affinity maturation required to obtain neutralizing HIV-1 antibodies is one of the main reasons why the structure-based reverse vaccinology approach did not succeed in developing an effective HIV-1 vaccine (3). A new paradigm based on the germline/maturation hypothesis was subsequently developed, which assumed that it may be possible to discover effective HIV-1 vaccine immunogens by analyzing putative germline antibody intermediates of known HIV-1 nMabs (13). It is not clear at present whether the unraveling of large numbers of different antibody maturation pathways will allow the identification of HIV-1 vaccine immunogens suitable for vaccinating large human populations.

## Assumption No. 4: The so-called Rational Design of HIV-1 Immunogens is More Effective than the Classical Trial-and-Error Screening of Immunogens used in the Past for Developing Successful Vaccines

Many authors claim that so-called “rational design” offers the best prospects for developing an HIV-1 vaccine. Design means the deliberate conceiving of a novel object or process by an intelligent being, while the term “rational” is mostly used to indicate that the designer makes use of available molecular data (8, 14). When they discuss the rational design of HIV-1 vaccines, these authors refer only to studies that try to improve the structural complementarity between one epitope and one particular Mab, which means they actually discuss antigen design and not immunogen design (15, 16). Designing vaccine immunogenicity actually means modifying an antigen, so that it becomes an immunogen capable of inducing a protective immune response and this requires investigating empirically the numerous factors, which in an immunized host determine the formation of neutralizing antibodies. Unfortunately, we know very little about the immunological mechanisms in the host that produce neutralizing rather than non-neutralizing antibodies and our ignorance is what prevents us from deliberately “designing” vaccine immunogens of predetermined, known efficacy. Rationally designing an antigen so that it better fits a single Mab is certainly possible but improving an immunogen so that it is better able to elicit protective antibodies can only be achieved by trial-and-error experimentation with various immunogens and not by design (8, 17). It is currently fashionable to denigrate empirical approaches in vaccine research (18), as if time-honored empiricism and trial-and-error experimentation were not an entirely rational enterprise compatible with high quality science (3, 7, 14). Advocating rational design instead of stressing the need for more empirical research may even be counterproductive since it is likely to make it more difficult for investigators to undertake the small exploratory trial-and-error trials that are needed to move the field forward (5). Although epitope mapping with nMabs may indicate which regions of a virus surface should be targeted by a vaccine, such mapping on its own will not make it possible to discover effective vaccine immunogens if no immunogenicity trials are performed.

## Assumption No. 5: Reactions of Viral Antigens with Mabs are More Specific than the Combined Reactivity of Polyclonal Antibodies Found in an Antiserum and are able to Inform Vaccine Design

The introduction of Mabs revolutionized our ability to dissect immune responses to proteins but it also introduced a bias in the analysis of antigens by encouraging investigators to focus on artificial boundaries between overlapping epitopes and to concentrate on single, discrete epitopes as potential vaccine immunogens. The emphasis on single epitopes recognized by Mabs for developing vaccines tends to obscure the beneficial effect of the neutralizing synergy achievable with polyclonal responses (19).

The use of Mabs for characterizing epitopes also introduced another bias that occurs because the apparent specificity of a Mab very much depends on the selection process that was used to obtain it. When a nMab, for instance, binds to a short peptide region of the membrane proximate external region (MPER) of HIV-1 gp41, this may simply reflect the fact that the Mab was selected for its ability to bind to that peptide. Since all antibodies are polyspecific, the Mab may actually recognize better a more complex or transient epitope of gp41 that might have been the immunogen that elicited the antibody. However, if it is assumed that the Mab was induced by the linear MPER peptide region because it reacts with it, searching for an effective vaccine immunogen in gp41 may be compromised.

It is often believed that a Mab that binds to a given antigen is a more specific reagent than a polyclonal antiserum raised against that antigen. In reality, an antiserum has a greater collective specificity for a multiepitopic antigen due to the additive specificity effect that arises from the presence in the antiserum of antibodies directed to several different epitopes of the antigen (20).

It is sometimes suggested that the isolation of additional broadly neutralizing Mabs will facilitate the future rational design of an HIV-1 vaccine. Why this should be the case is not made clear since these Mabs only have the potential to perhaps 1 day become useful reagents for *passive* immunotherapy. However, their isolation does not improve our ability to discover which vaccine immunogens will elicit neutralizing antibodies by means of *active* immunization (14). Once again, confounding antigenicity and immunogenicity is giving rise to unrealistic expectations.

The aim of this Research Topic is to evaluate the shortcomings of some of the paradigms that guided HIV vaccine research in the past as well as to stimulate the search for novel paradigms that better fit our current understanding of immunological specificity and could be more helpful in guiding the future search for an effective HIV-1 vaccine.

## Conflict of Interest Statement

The author declares that the research was conducted in the absence of any commercial or financial relationships that could be construed as a potential conflict of interest.
